# Efficacy and safety of rivaroxaban versus warfarin in the management of unusual site deep vein thrombosis: a retrospective cohort study

**DOI:** 10.3389/fphar.2024.1419985

**Published:** 2024-06-12

**Authors:** Linlin Fu, Wenting Cai, Hanyang Li, Dan Han, Li Li, Baoyan Wang

**Affiliations:** ^1^ Department of Pharmacy, Nanjing Drum Tower Hospital, Basic Medicine and Clinical Pharmacy College, China Pharmaceutical University, Nanjing, China; ^2^ Department of Biochemistry and Molecular Biology, Nanjing Medical University, Nanjing, China; ^3^ Department of Pharmacy, Nanjing Drum Tower Hospital, Affiliated Hospital of Medical School, Nanjing University, Nanjing, China

**Keywords:** rivaroxaban, warfarin, unusual site DVT, efficacy, safety

## Abstract

**Background:**

Unusual site deep vein thrombosis (DVT) was defined as venous thromboembolism (VTE) occurring outside the conventional deep veins of the lower extremity or pulmonary arteries. However, the optimal anticoagulation therapy for unusual site DVT remained unclear. This study aims to evaluate the efficacy and safety of rivaroxaban in unusual site DVT.

**Methods:**

This retrospective cohort study enrolled consecutive patients at Nanjing Drum Tower Hospital between January 2011 and December 2021 who were diagnosed with unusual site DVT. Patients were divided into two groups based on their ultimate medication choice: the warfarin group and the rivaroxaban group. The demographic characteristics were recorded for all enrolled patients. Clinical outcomes included recurrent VTE, bleeding complications and major bleeding.

**Results:**

A total of 1,088 patients were divided into warfarin (*n* = 514) and rivaroxaban (*n* = 574) groups. After the stabilized inverse probability of treatment weighting, Hazard Ratios for warfarin vs. rivaroxaban of recurrent VTE, bleeding complications and major bleeding were 0.52(95% CI: 0.25–1.08), 0.30(95% CI: 0.14–0.60), and 0.33 (95% CI, 0.13–0.74), respectively. Risk of clinical outcomes in specified subgroups for age, gender, renal function, thrombosis sites and diagnosis were assessed. The interaction of gender and treatment on major bleeding was significant (P for interaction = 0.062). Otherwise, there was no significant interaction between the other subgroups and the treatment group in terms of clinical outcomes.

**Conclusion:**

Compared with warfarin, rivaroxaban exhibited comparable efficacy for the anticoagulant treatment of unusual site DVT, associated with a lower risk of bleeding complications and major bleeding.

## 1 Introduction

Venous thromboembolism (VTE) usually occurred in the lower extremity and pulmonary arteries; however, it can also occur in other veins, which we call unusual site Deep Vein Thrombosis (DVT). Common unusual site DVT, such as splanchnic vein thrombosis (SVT), upper extremity deep vein thrombosis (UEDVT) and cerebral venous thrombosis (CVT), collectively represent approximately 10% of all venous thrombosis cases, yet exhibiting a higher mortality rate (Abbattista et al., 2020). Although less prevalent than lower extremity DVT (LEDVT), the incidence of unusual site DVT was increasing, attributed to the increased frequency of risk factors. The clinical manifestation of unusual site DVT was marked by heterogeneity, contingent upon the specific vein affected, associated risk factors, and the local or systemic clinical context (Di Nisio et al., 2020). Patients with unusual site DVT faced an elevated risk of bleeding compared to those with common VTE, and the potential for recurrent VTE cannot be disregarded (Di Nisio et al., 2020). However, the level of evidence and recommendation in the currently available guidelines were low, and developing overall recommendations for the clinical management of unusual sites DVT remained a daunting challenge.

Anticoagulant therapy was a frequently contemplated therapeutic approach for patients newly diagnosed unusual site DVT (Tufano et al., 2018; Valeriani et al., 2019). Conventional anticoagulant therapy involved the application of warfarin and low molecular weight heparin (LMWH) in those patients (Stevens et al., 2021). The currently recommended anticoagulant therapy for unusual site VTE was rooted in parenteral anticoagulation with LMWH, followed by warfarin (Ferro et al., 2017; European Association for the Study of the Liver. Electronic address: easloffice@easloffice.eu, 2016). In recent years, the efficacy and safety of non-vitamin K promising antagonist oral anticoagulants (NOACs) in treating LEDVT had received comprehensive validation, leading to their increasingly prevalent use in clinical practice (Riva and Ageno, 2015). Among these NOACs, rivaroxaban standed out as the most extensively utilized anticoagulant medication. While rivaroxaban had solidified its role as a first-line treatment for pulmonary embolism (PE) and DVT, its application in unusual site DVT remains relatively unexplored (Chopard et al., 2020).

Presently, there were limited available data on unusual site DVT. Racial differences existed in the incidence, age of onset and gender distribution of unusual site DVT (Li et al., 2019), resulting in a dearth of comprehensive data that reduced the precision and representativeness of the treated population (Li et al., 2019). Consequently, the present study served as a retrospective analysis aimed at meticulously assessing the efficacy and safety associated with the use of rivaroxaban for the treatment of unusual site DVT.

## 2 Materials and methods

### 2.1 Study design and patients

This retrospective cohort study enrolled consecutive patients diagnosed with unusual site DVT in Nanjing Drum Tower Hospital between January 2011 and December 2021. Approval for the study was obtained from the Institutional Ethics Committees of Nanjing Drum Tower Hospital (Approval Number: 2023-589-029), and the study protocol adhered to the ethical principles outlined in the Declaration of Helsinki.

The diagnosis of unusual site DVT was confirmed by using venography or Doppler ultrasonography. The patients not receiving anticoagulant therapy, receiving antithrombotic drugs other than warfarin and rivaroxaban (e.g., aspirin, clopidogrel, or other NOACs), combined with diseases requiring long-term anticoagulant therapy (e.g., atrial fibrillation, atrial flutter or valvular heart disease), with severe hepatic or renal insufficiency (classified as Child-Pugh class B or C, or creatinine clearance <15 mL/min) and with a history of thrombosis were excluded from the analysis.

The anticoagulant therapy was carefully reviewed and recorded. Considering the potential alterations in medications during the course of anticoagulant therapy, patients were divided into two groups based on their ultimate medication choice: the warfarin group and the rivaroxaban group. The duration of anticoagulation therapy was maintained for at least 3 months. The initiation of the follow-up period coincided with the time when the patient started anticoagulant therapy with warfarin or rivaroxaban and lasted until the occurrence of clinical outcomes or the end date of the study period (31 Dec 2022). The clinical outcomes of patients were obtained from subsequent outpatient or inpatient medical records. Incomplete follow-up information was overcome with contacting patients by telephone.

### 2.2 Covariates

The demographic characteristics, including age, gender, body mass index (BMI), laboratory indexes, combined diseases, thrombosis sites and the types of thrombosis, were recorded for all enrolled patients. Renal function was categorized based on creatinine clearance (CrCl), distinguishing between normal renal function (CrCl >90 mL/min) and abnormal renal function (CrCl 15–90 mL/min). The investigation specifically focused on three types of unusual site DVT: SVT, CVT, and UEDVT. SVT included non-cirrhotic portal vein thrombosis, splenic vein thrombosis, mesenteric vein thrombosis and Budd-Chiari syndrome (BCS). CVT included dural venous sinus thrombosis, cortical vein thrombosis and deep cerebral vein thrombosis. The locations of UEDVT involved the brachial vein, axillary vein, subclavian vein, cephalic arm vein, radial vein, ulnar vein and internal jugular vein. All covariates were adjusted using inverse probability weighting (IPTW).

### 2.3 Study outcome

The efficacy outcome was the occurrence of recurrent VTE, defined as follows: confirmed thrombosis propagation, occurrence of a new DVT unrelated to the initial DVT or new PE confirmed by pulmonary angiography. Thrombosis propagation indicated the extension of thrombosis within the initial venous segment and beyond its initial length, or its expansion into a new venous segment. The diagnostic criteria for DVT involved ultrasound evidence of noncompressible venous segments or venography demonstration of intraluminal filling defects. Patients with non-recanalized thrombosis were defined as persistent occlusion of a previously occluded vein segment on imaging, with no change in the extent of thrombosis compared with pretreatment imaging.

The safety outcome included all bleeding complications, defined by the International Society of Thrombosis and Haemostasis (ISTH). Major bleeding was characterized as fatal bleeding, bleeding from critical areas or organs (e.g., intracranial, spinal, pericardial, articular, retroperitoneal), bleeding resulting in a hemoglobin drop of >2 g/dL, or bleeding necessitating the transfusion of >2 units of whole blood or packed red blood cells. Clinically relevant non-major (CRNM) bleeding was defined as overt hemorrhage that did not meet the criteria for major bleeding but necessitated medical intervention, unscheduled consultation with a physician, discontinuation of medication or disruption of routine daily activities.

### 2.4 Statistical analysis

To compare clinical outcomes between the warfarin and rivaroxaban groups, stabilized inverse probability of treatment weights (IPTW) method was used to balance the covariates to ensure comparability of results. Propensity scores of each treatment group were computed through multivariate logistic regression of all covariates. Then stabilized inverse probability of treatment weights based on the propensity scores was used to adjust for measured covariates, which created a pseudo dataset by preserving sample size. To assess the performances before and after stabilized IPTW, we compared the covariates using standardized mean differences (SMD), with differences >10% regarded as imbalanced. The weighted incidence rate (IR) was determined by dividing the weighted number of clinical outcomes by 100 person-years. Kaplan-Meier method assessed the differences of clinical outcomes between different groups, using Log-Rank test for survival curve difference. Hazard ratios (HRs) were calculated using the multivariate Cox proportional risk model, with the warfarin group as reference.

Among the data set of total 1,088 patients, missing data on BMI, CrCl, platelet, fibrinogen and hemoglobin existed in 64 patients (5.88%) and were imputed using the multiple imputation of chained equations. We used the R statistical software to create five imputed datasets and pool the HR values. Complete data was utilized for subsequent statistical analysis. *p*-value <0.05 was considered significant.

Risk of clinical outcomes in specified subgroups (age, gender, renal function, number of thrombosis sites and diagnosis) was assessed. For subgroup analysis, multivariate Cox proportional hazards regression and propensity score methods were employed. The significance of interaction between treatment group and subgroup was defined as P-for-interaction <0.1.

## 3 Results

### 3.1 Patient characteristics

Between 2011 and 2021, a total of 1,088 patients were enrolled in the study, categorized into two treatment groups: 574 patients receiving warfarin and 514 patients receiving rivaroxaban. Prior to IPTW, notable imbalances of baseline characteristics existed (Table1). The rivaroxaban group exhibited a higher representation of male patients and an elevated mean BMI compared with the warfarin group. Furthermore, patients in the rivaroxaban group displayed higher platelet and fibrinogen level but lower hemoglobin level. Rivaroxaban therapy was more frequently administered to patients with systemic diseases such as hypertension, cancers, diabetes and cerebral hemorrhage. Patients with a single thrombosis site were inclined to be in the warfarin group. Regarding the diagnosis of unusual site DVT, the rivaroxaban group had a lower proportion of patients with SVT and CVT. Following IPTW weighting, all baseline characteristics were effectively balanced in both treatment groups, ensuring a more reliable comparative analysis.

**TABLE 1:** Baseline characteristics of patients received warfarin vs. rivaroxaban before and after inverse probability of treatment weighting (IPTW).

	Before IPTW			After IPTW		
	Warfarin (N = 514)	Rivaroxaban (N = 574)	SMD	Warfarin (N = 531)	Rivaroxaban (N = 570)	SMD
Gender (%)						
Male	294 (57.20)	360 (62.72)	0.113	324 (61.10)	346 (60.70)	0.008
Female	220 (42.80)	214 (37.28)		207 (38.90)	224 (39.30)	
Age	59.42 ± 15.84	58.82 ± 15.71	0.038	59.27 ± 15.55	59.33 ± 15.55	0.007
BMI	22.68 ± 3.24	23.41 ± 3.61	0.212	23.13 ± 3.50	23.01 ± 3.50	0.036
Laboratory examinations						
Platelet Count (×10^9^/L)	161.61 ± 110.41	181.84 ± 125.03	0.172	170.39 ± 120.43	170.77 ± 115.99	0.003
Fibrinogen (g/L)	2.61 ± 1.00	2.96 ± 1.32	0.295	2.70 ± 0.99	2.77 ± 1.26	0.057
Hemoglobin (g/L)	120.55 ± 31.14	112.54 ± 28.61	0.268	116.56 ± 30.18	115.75 ± 29.67	0.027
Renal function (%)						
Normal	373 (72.57)	392 (68.29)	0.094	361 (68.00)	396 (69.47)	0.030
Abnormal	141 (27.43)	182 (31.71)		170 (32.00)	174 (30.53)	
Systemic diseases (%)						
Hypertension	122 (23.74)	168 (29.27)	0.126	152 (28.63)	150 (26.32)	0.052
Inflammation	135 (26.26)	133 (23.17)	0.072	122 (22.98)	150 (26.32)	0.078
Cancers	27 (5.25)	158 (27.53)	0.631	104 (19.59)	98 (17.19)	0.064
Diabetes	49 (9.53)	88 (15.33)	0.176	65 (12.24)	72 (12.63)	0.01
Dyslipidemia	12 (2.33)	18 (3.14)	0.049	11 (2.07)	15 (2.63)	0.039
Cerebral Hemorrhage	37 (7.20)	67 (11.67)	0.154	58 (10.92)	56 (9.82)	0.042
Coronary Artery Diseases	14 (2.72)	17 (2.96)	0.014	12 (2.26)	15 (2.63)	0.02
Pregnancy	10 (1.95)	19 (3.31)	0.085	15 (2.82)	16 (2.81)	<0.001
Autoimmune diseases	28 (5.45)	31 (5.40)	0.002	31 (5.84)	32 (5.61)	0.008
Thrombophilia	3 (0.58)	6 (1.05)	0.051	4 (0.75)	5 (0.88)	0.014
Thrombosis sites						
1	404 (78.60)	404 (70.38)	0.189	365 (68.74)	418 (73.33)	0.100
≥2	110 (21.40)	170 (29.62)		166 (31.26)	152 (26.67)	
Diagnose (%)						
Splanchnic Vein Thrombosis	376 (73.15)	340 (59.23)	0.485	342 (64.41)	379 (66.49)	0.086
Cerebral Venous Thrombosis	107 (20.82)	105 (18.30)		93 (17.51)	106 (18.60)	
Upper Extremity Deep Vein Thrombosis	31 (6.03)	129 (22.47)		96 (18.08)	85 (14.91)	

### 3.2 Clinical outcomes

**FIGURE 1:**
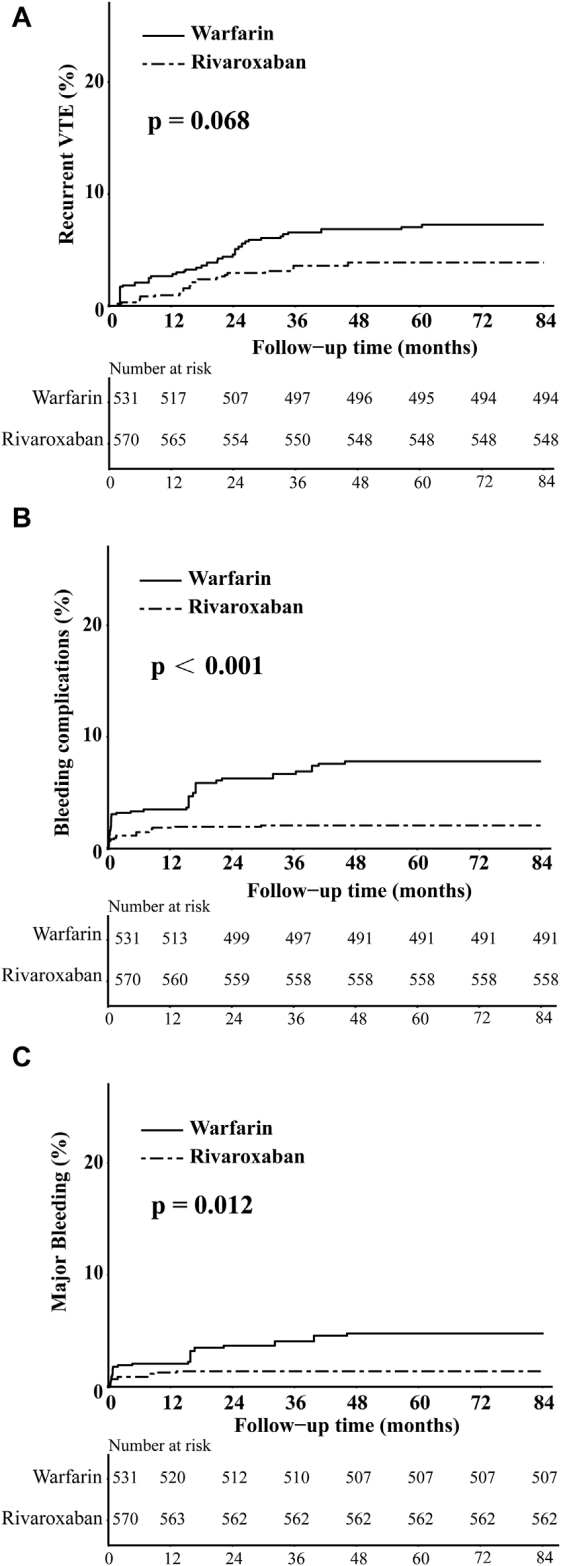
The weighted cumulative incidence curves of recurrent VTE **(A)**, bleeding complications **(B)**, and major bleeding **(C)** in warfarin and rivaroxaban groups.

**FIGURE 2:**
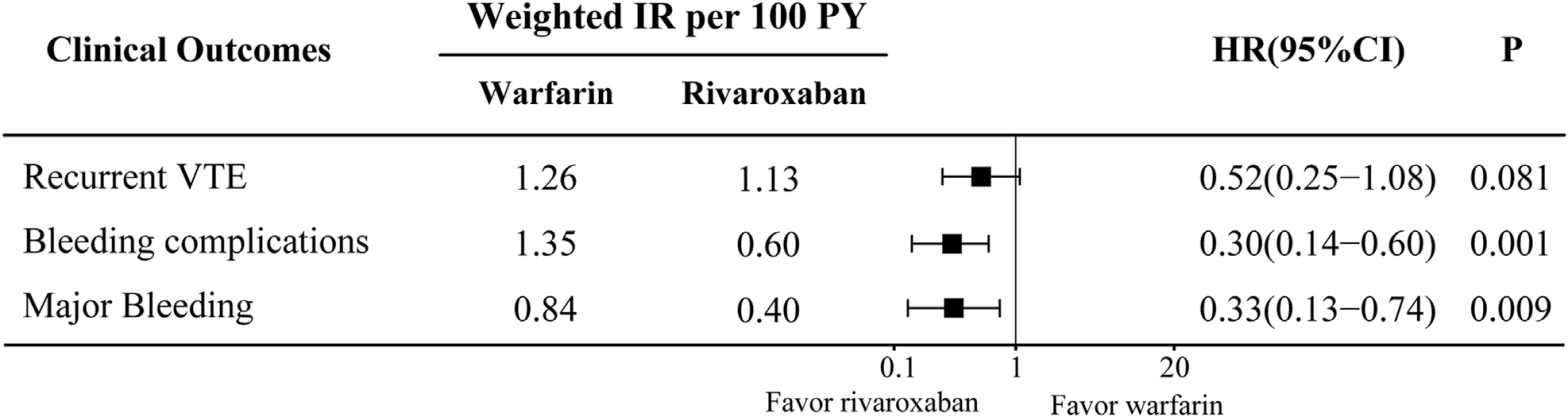
Weighted incidence rates (IRs) and hazard ratios (HRs) of clinical outcomes compare between warfarin and rivaroxaban groups. CI, Confidence interval.

In the comparison between the warfarin and rivaroxaban groups, the incidences of recurrent VTE, bleeding complications, and major bleeding were 5.84% vs. 3.89%, 6.81% vs. 2.53%, and 4.09% vs. 1.75%, respectively. Visual representations of the weighted cumulative incidence curve, weighted IRs, and HRs for clinical outcomes were showed in Figures 1, 2. No significant difference was observed in the occurrence of recurrent VTE between the two groups (HR: 0.52, 95% CI: 0.25–1.08). In comparison to warfarin, rivaroxaban exhibited a noteworthy reduction in the risk of bleeding complications (HR: 0.30, 95% CI: 0.14–0.60) and significantly diminish the risk of major bleeding (HR: 0.33, 95% CI: 0.13–0.74).

Additionally, the incidence of non-recanalization during the follow-up period was recorded, 10.89% in the warfarin group and 13.06% in the rivaroxaban group. Subsequent logistic regression analysis indicated a similar risk of incomplete recanalization of thrombosis between the two groups (OR: 1.04, 95% CI: 0.99–1.08).

### 3.3 Subgroup analysis

**FIGURE 3:**
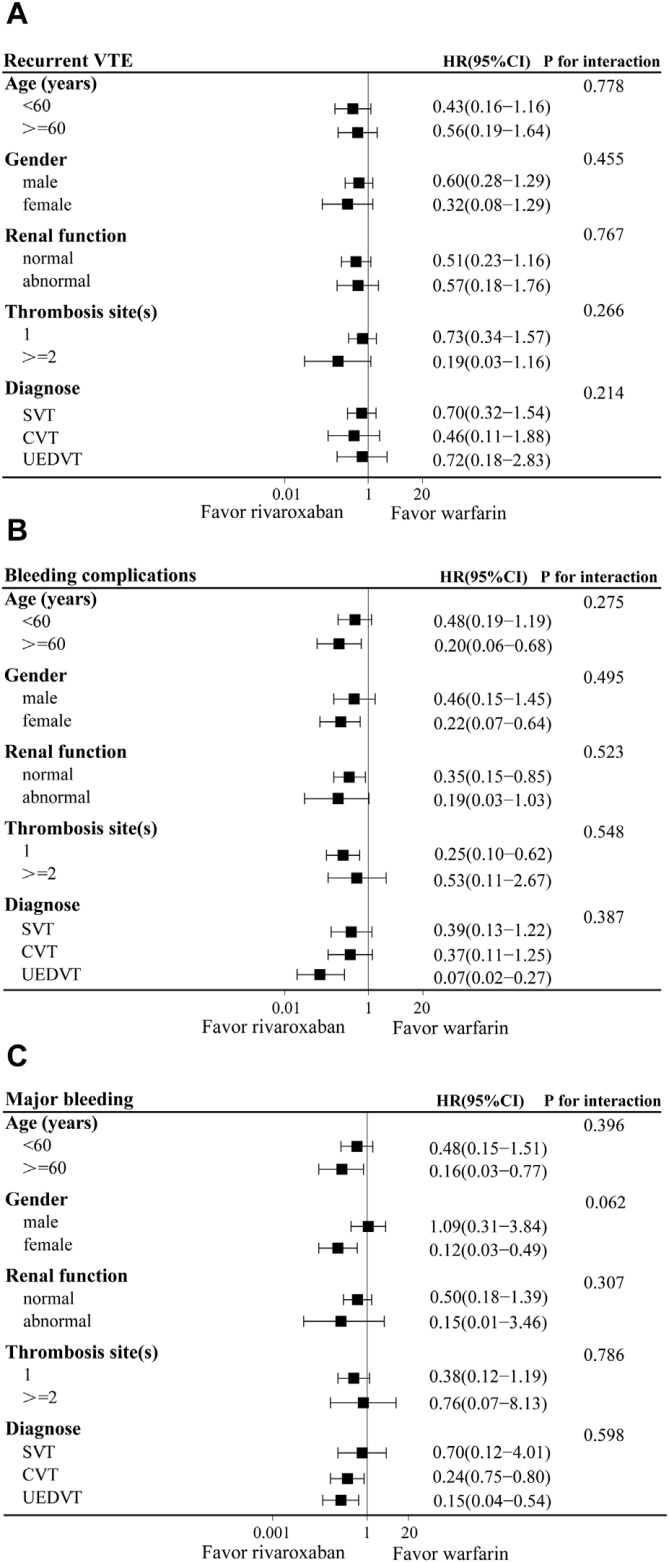
Adjusted hazard ratios (HRs) of recurrent VTE **(A)**, bleeding complications **(B)**, and major bleeding **(C)** according to various subgroups in warfarin and rivaroxaban groups. CI, Confidence interval; SVT, splanchnic vein thrombosis; UEDVT, upper extremity deep vein thrombosis; CVT, cerebral venous thrombosis.

The results of the subgroup analysis were shown in Figure 3. Demographic data showed that 42.4% and 47.2% of patients were younger than 60 years in the warfarin and rivaroxaban groups, respectively. Using 60 years as the cutoff to define age subgroups, rivaroxaban reduced the risk of bleeding complications and major bleeding compared with the warfarin group in patients over 60 years old (HR: 0.20, 95% CI: 0.06–0.68; HR: 0.16, 95% CI: 0.03–0.77), but the interaction between age and treatment group was not significant. In terms of major bleeding, a significant interaction between the gender and treatment groups indicated that rivaroxaban was associated with a lower risk of major bleeding in female patients (HR: 0.12, 95% CI: 0.03–0.49; P for interaction 0.062). For the renal function, thrombosis sites and diagnose stratification, there were no significant interactions between the subgroups and treatment groups in the recurrent VTE. Although rivaroxaban reduced the risk of bleeding complications in patients with normal renal function or UEDVT, and the risk of major bleeding in patients with CVT or UEDVT, no significant interactions between subgroups and treatment groups were observed. Therefore, the results of the subgroup analysis were basically consistent with the total study population.

## 4 Discussion

The management of unusual site DVT typically relied on anticoagulation protocols recommended for patients with common sites of VTE. Nevertheless, the distinct sites of thrombosis and specific risk factors associated with unusual site DVT necessitated a nuanced approach. Given the complexity of conditions, the application of these anticoagulant medications required a meticulous evaluation of the risk of recurrent VTE, bleeding complications, and both short- and long-term outcomes. The absence of guidelines with robust evidence and recommendations had resulted in a lack of clinical management therapies for unusual site DVT in real-world medical practice. In this retrospective cohort study, we assessed the safety and efficacy of rivaroxaban and warfarin in patients diagnosed with unusual site DVT. Our findings indicated that both rivaroxaban and warfarin demonstrated a similar risk of recurrent VTE. However, rivaroxaban exhibited a lower risk of bleeding complications and major bleeding. Rivaroxaban had been reported to be as effective and safe as warfarin in the treatment of VTE and is highly effective in preventing recurrent VTE with an acceptable risk of bleeding if treatment was continued (Bauersachs et al., 2010). Although the pathogenesis, susceptibility factors, clinical manifestations, and prognosis of unusual site DVT are different from LEDVT, the results of our study regarding the use of rivaroxaban are similar to those in LEDVT.

This study investigated three common types of unusual site DVT, including SVT, CVT and UEDVT, which were further analyzed in the subgroup analysis. SVT encompassed thrombotic events occurring in the visceral venous circulation, involving the portal, mesenteric, splenic veins, and BCS (Di Nisio et al., 2020). SVT had a higher risk of bleeding complications compared to common sites of VTE, and in addition, the risk of thrombotic recurrence cannot be ignored (Ageno et al., 2017). According to guidelines from the International Society on Thrombosis and Haemostasis (ISTH), NOACs are recommended for patients with acute non-cirrhotic SVT (Di Nisio et al., 2020). However, the American College of Gastroenterology guidelines, citing limited experience with NOACs, recommended initiating anticoagulation with parenteral anticoagulation, followed by warfarin (Simonetto et al., 2020). Our study revealed that the risks of recurrent VTE, bleeding complications and major bleeding were analogous between rivaroxaban and warfarin in patients with SVT. An interventional prospective cohort study enrolling 100 patients with non-cirrhotic SVT treated with rivaroxaban, reported 2 major bleeding and 2 recurrent cancer-related VTE in patients with potential predisposing factors, suggesting that rivaroxaban was safe and effective in the treatment of non-cirrhotic SVT (Ageno et al., 2022). Current studies have prompted clinicians to prescribe rivaroxaban for patients with SVT.

CVT constituted a spectrum of vascular disorders arising from either partial or complete occlusion of cerebral venous sinuses, or smaller cortical veins. Partial or complete occlusion of vein resulted in a rapid surge in intracranial pressure, potentially leading to severe bleeding or fatal outcomes (Capecchi et al., 2018). The American Heart Association (AHA) guidelines for stroke prevention stated that anticoagulation was indicated for patients with CVT accompanied by intracranial hemorrhage (Kernan et al., 2014). However, the choice of anticoagulant medications should take into account the recurrent long-term intracranial hemorrhage. The European Stroke Organization (ESO) guidelines (2017) cautiously advocated for parenteral anticoagulation followed by warfarin in CVT patients, while discouraging the use of NOACs. However, this recommendation was considered weak due to the very low quality of evidence available. A multicenter observational study involving 111 patients with CVT, showing the similar efficacy and lower incidence of major bleeding of NOACs compared with warfarin (Wasay et al., 2019). Our study also yielded the same results. Rivaroxaban had been reported to reduce the risk of major bleeding by at least 50%, and its safety benefit was particularly important for CVT patients, as one-third of CVT patients experienced cerebral hemorrhage (van Es et al., 2014).

UEDVT usually originated in the subclavian, axillary and brachial veins, and may extend to the cephalic, brachial veins and even the superior vena cava (Bosch et al., 2020). Its incidence had been increasing over the years, particularly due to the rising incidence of cancers, and the increased utilization of indwelling central venous catheters (CVCs), pacemakers and defibrillators (Ageno et al., 2019). UEDVT had a lower incidence of concomitant PE, recurrent VTE and post-thrombotic syndrome compared to LEDVT, but in terms of mortality was higher in patients with UEDVT, which may reflect the higher prevalence and severity of cancers (Riva and Ageno, 2021). Patients with cancer-related UEDVT account for 40% of all UEDVT patients and were the focus of specific studies, while cancer-related UEDVT patients accounted for 53% of UEDVT patients in our study (Bleker et al., 2016). A systematic evaluation showed that patients with cancer-related UEDVT had a 2-3 times higher risk of recurrent VTE and a 4 times higher risk of bleeding complications than patients without cancer (Bleker et al., 2016). Our study found that the rivaroxaban group had similar recurrent VTE with a lower risk of bleeding complications and major bleeding compared to the warfarin group. The efficacy and safety advantages of rivaroxaban may provide significant benefits for cancer-related UEDVT.

The subgroup analysis in this study indicated that rivaroxaban was linked to a diminished risk of major bleeding in female patients. In a meta-analysis examining gender differences in NOACs treatment, the results indicated a trend toward an elevated risk of major bleeding in male patients compared to female patients (Dentali et al., 2015). The risk of major bleeding associated with rivaroxaban exhibited gender-specific patterns. Despite the reduced risk of major bleeding, certain reports suggested that young female patients may encounter increased menstrual bleeding intensity with rivaroxaban (Bryk et al., 2016).

This study had certain limitations that should be acknowledged. Firstly, being a retrospective cohort study, the efficacy and safety outcomes may have been influenced by undisclosed variables inherent to retrospective designs, despite efforts to mitigate such influences through IPTW methods to achieve balance in differences between group. Secondly, this was a retrospective study with a limited number of patients included, which may have weakened the reliability of this study. Lastly, the inclusion of patients diagnosed between 2011 and 2021 may introduce a temporal bias, as clinicians increasingly prescribed rivaroxaban since 2015. Temporal shifts in diagnostic and therapeutic approaches during the study period may have affected the results.

## 5 Conclusion

In summary, the available evidence supported the utilization of rivaroxaban for the treatment of patients with unusual site DVT. Rivaroxaban demonstrated efficacy comparable to warfarin while exhibiting a more favorable safety profile. The current landscape challenged the conventional warfarin therapy, advocating for the increased adoption of rivaroxaban in patients with unusual site DVT, given its demonstrated safety, efficacy, therapeutic efficiency, and simplicity. Further research and clinical trials are imperative to augment the level of evidence in this field in the future.

## Data Availability

The raw data supporting the conclusion of this article will be made available by the authors, without undue reservation.
