# Supplementary material for: Efficacy and safety of rivaroxaban versus warfarin in the management of unusual site deep vein thrombosis: a retrospective cohort study
Source: Front Pharmacol. 2024 Jun 12;15:1419985. doi: 10.3389/fphar.2024.1419985 (PMC11199520; doi:10.3389/fphar.2024.1419985)

***Supplementary Material***

Missing data for BMI and renal function, constituting 5.9% (64 of 1088) of total data items, underwent imputation using multiple imputation of chained equations with baseline characteristics. Five imputed datasets were generated and outcomes were pooled. Additionally, sensitivity analysis was conducted using complete data. It is important to note that both subgroup and sensitivity analyses are exploratory and susceptible to type I errors due to multiple comparisons.

Post IPTW, all baseline information was effectively balanced between the two groups. Weighted IRs and HRs for clinical outcomes from sensitivity analyses are depicted in Figures 4. No significant difference in recurrent VTE was observed between the two groups (HR, 0.51; 95% CI, 0.24-1.08). In comparison to warfarin, rivaroxaban demonstrated a reduced risk of bleeding and major bleeding (HR, 0.29; 95% CI, 0.14-0.60 and HR, 0.30; 95% CI, 0.12-0.71). Logistic regression analysis revealed a similar risk of non-recanalization of thrombosis between the two groups (OR, 1.04; 95% CI, 0.99-1.08).

Supplementary Table S1. Baseline characteristics of patients received warfarin vs rivaroxaban before and after inverse probability of treatment weighting (IPTW) in sensitivity analysis.

|  | **Before IPTW** | | | **After IPTW** | | |
| --- | --- | --- | --- | --- | --- | --- |
|  | **Warfarin**  **(N=478)** | **Rivaroxaban (N=546)** | **SMD** | **Warfarin**  **(N=493)** | **Rivaroxaban (N=542)** | **SMD** |
| **Gender (n, (%))** |  |  |  |  |  |  |
| Male | 274(57.32) | 347(63.55) | 0.128 | 304(61.66) | 333(61.44) | 0.008 |
| Female | 204(42.67) | 199(36.45) |  | 189(38.34) | 209(38.56) |  |
| **Age (years)** | 59.90±15.89 | 58.91±15.59 | 0.063 | 59.39±15.77 | 59.62±15.51 | 0.015 |
| **BMI** | 22.73±3.26 | 23.43±3.62 | 0.202 | 23.21±3.59 | 23.04±3.51 | 0.048 |
| **Laboratory Examinations** |  |  |  |  |  |  |
| Platelet Count | 161.16±111.49 | 178.31±109.71 | 0.155 | 169.16±120.60 | 168.59±106.24 | 0.005 |
| Fibrinogen | 2.63±1.01 | 2.96±1.33 | 0.277 | 2.71±1.00 | 2.78±1.27 | 0.059 |
| hemoglobin | 122.20±30.50 | 112.75±28.48 | 0.320 | 117.57±29.80 | 116.70±29.54 | 0.029 |
| **Renal Function (n, (%))** |  |  |  |  |  |  |
| normal | 355(74.27) | 384(70.33) | 0.088 | 343(69.57) | 387(71.40) | 0.039 |
| abnormal | 123(25.73) | 162(29.67) |  | 150(30.43) | 155(28.60) |  |
| **Systemic diseases (n, (%))** |  |  |  |  |  |  |
| Hypertension | 118(24.69) | 163(29.85) | 0.116 | 144(29.21) | 147(27.12) | 0.048 |
| Inflammation | 123(25.73) | 125(22.89) | 0.066 | 111(22.52) | 140(25.83) | 0.076 |
| Cancers | 26(5.43) | 151(27.66) | 0.626 | 95(19.27) | 95(17.53) | 0.047 |
| Diabetes | 48(10.04) | 86(15.75) | 0.171 | 62(12.58) | 72(13.28) | 0.018 |
| Dyslipidemia | 11(2.30) | 18(3.30) | 0.060 | 10(2.03) | 15(2.77) | 0.042 |
| Cerebral Hemorrhage | 34(7.11) | 64(11.72) | 0.158 | 57(11.56) | 53(9.78) | 0.058 |
| Coronary Artery Diseases | 14(2.93) | 17(3.11) | 0.011 | 12(2.43) | 15(2.77) | 0.016 |
| Pregnancy | 6(1.26) | 16(2.93) | 0.117 | 13(2.64) | 12(2.21) | 0.026 |
| Autoimmune Diseases | 25(5.23) | 30(5.49) | 0.012 | 29(5.88) | 30(5.53) | 0.015 |
| Thrombophilia | 3(0.63) | 4(0.73) | 0.013 | 4(0.81) | 4(0.74) | 0.007 |
| **Thrombosis site(s)** |  |  |  |  |  |  |
| 1 | 373(78.03) | 383(70.15) | 0.181 | 338(68.56) | 396(73.06) | 0.096 |
| ≥2 | 105(21.97) | 163(29.85) |  | 155(31.44) | 146(26.94) |  |
| **Diagnose (n, (%))** |  |  |  |  |  |  |
| Splanchnic Vein Thrombosis | 353(73.85) | 324(59.34) | 0.471 | 318(64.50) | 361(66.61) | 0.078 |
| Cerebral Venous Thrombosis | 94(19.67) | 99(18.13) |  | 86(17.44) | 99(18.27) |  |
| Upper Extremity Deep Vein Thrombosis | 31(6.48) | 123(22.53) |  | 89(18.06) | 82(15.12) |  |

Supplementary Figure S1. Weighted incidence rates (IRs) and hazard ratios (HRs) of clinical outcomes compare between warfarin and rivaroxaban groups in sensitivity analysis. CI, Confidence interval.


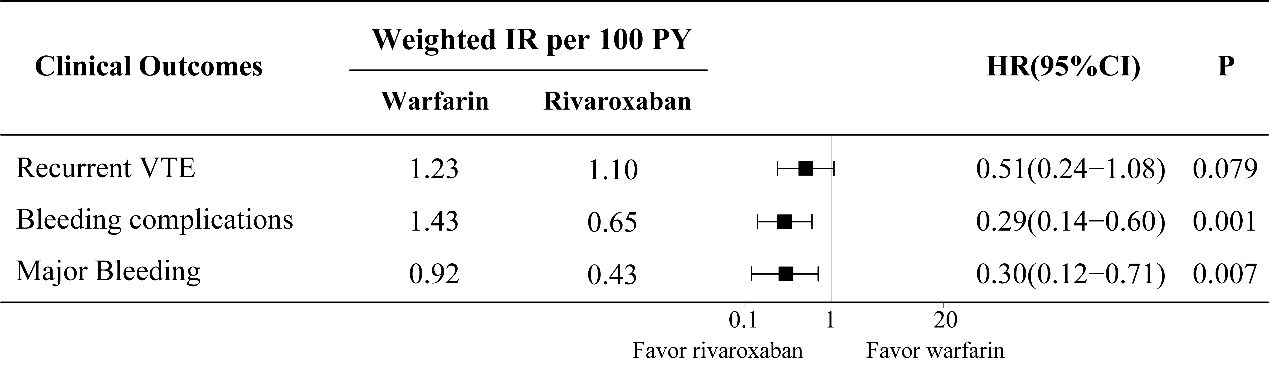

Supplement: Supplementary file 1 [file DataSheet1.docx]
